# *Afropectinariella* (Vandeae, Orchidaceae), a new genus of the *Angraecum* alliance

**DOI:** 10.3897/phytokeys.96.23933

**Published:** 2018-04-13

**Authors:** Murielle Simo-Droissart, Bonaventure Sonké, Vincent Droissart, Tariq Stévart

**Affiliations:** 1 Plant Systematics and Ecology Laboratory, Higher Teachers’ Training College, University of Yaoundé I, P.O. Box 047, Yaoundé, Cameroon; 2 Missouri Botanical Garden, Africa and Madagascar Department, P.O. Box 299, St. Louis, Missouri 63166-0299, USA; 3 Herbarium et Bibliothèque de Botanique africaine, C.P. 265, Université Libre de Bruxelles, Campus de la Plaine, Boulevard du Triomphe 1050, Brussels, Belgium; 4 AMAP, IRD, CIRAD, CNRS, INRA, Université de Montpellier, Montpellier, France; 5 Botanic Garden Meise, Domein van Bouchout, Nieuwelaan 38, B-1860 Meise, Belgium

**Keywords:** Angraecoid orchids, continental Africa, Malagasy and Indian Ocean islands, *Pectinaria*, phylogenetics, taxonomy

## Abstract

A recent phylogenetic study showed that species assigned to the newly recognised genus *Pectinariella* Szlach., Mytnik & Grochocka (previously treated as Angraecum Bory sect. Pectinaria Benth.) are polyphyletic, comprising a clade with species primarily in Madagascar and the Western Indian Ocean islands (including the type) and another non-sister clade whose members occur in continental Africa and the Gulf of Guinea islands. In order to render *Pectinariella* monophyletic, the five continental African species must therefore be removed. A new genus, *Afropectinariella* M.Simo & Stévart, is described and the following combinations are made: *Afropectinariella atlantica* (Stévart & Droissart) M.Simo & Stévart, *Afropectinariella doratophylla* (Summerh.) M.Simo & Stévart, *Afropectinariella gabonensis* (Summerh.) M.Simo & Stévart, *Afropectinariella pungens* (Schltr.) M.Simo & Stévart and *Afropectinariella subulata* (Lindl.) M.Simo & Stévart.

## Abstract (fr)

Une analyse phylogénétique récente a montré que les espèces attribuées au nouveau genre *Pectinariella* Szlach., Mytnik & Grochocka (anciennement Angraecum Bory sect. Pectinaria Benth.) forment un groupe polyphylétique, comprenant un clade avec des espèces présentes principalement à Madagascar et dans les îles de l'ouest de l'Océan Indien (incluant le type) et un autre clade qui ne lui est pas apparenté et dont les espèces sont distribuées en Afrique continentale et dans les îles du Golfe de Guinée. Afin de rétablir la monophylie du genre *Pectinariella*, les cinq espèces d’Afrique continentale et des îles du Golfe de Guinée doivent donc en être exclues. Un nouveau genre, *Afropectinariella* M.Simo & Stévart, est décrit et les combinaisons suivantes sont proposées: *Afropectinariella atlantica* (Stévart & Droissart) M.Simo & Stévart, *Afropectinariella doratophylla* (Summerh.) M.Simo & Stévart, *Afropectinariella gabonensis* (Summerh.) M.Simo & Stévart, *Afropectinariella pungens* (Schltr.) M.Simo & Stévart et *Afropectinariella subulata* (Lindl.) M.Simo & Stévart.

## Introduction

Since its description, the delimitation of *Angraecum* Bory (1804) has been controversial (Garay 1973). Much importance has been ascribed to the structure of the pollinia within *Angraecum*, which has led to the recognition of spurious genera to encompass closely related species (Schlechter 1913). Based on vegetative and floral features, along with geographic distribution, a number of partial or comprehensive revisions of the genus have been proposed over the decades (see Finet 1907; Garay 1973; Perrier de la Bathie 1941; Schlechter 1918, 1925; Senghas 1986; Stewart et al. 2006; Summerhayes 1958). Garay (1973) was the last to provide a full taxonomic treatment for *Angraecum*. He recognised 19 sections, of which nine are endemic to Madagascar and the Western Indian Ocean islands, one to Madagascar, the Western Indian Ocean islands and Sri Lanka, two to continental Africa and the Gulf of Guinea islands and seven have representatives in both Africa and Madagascar.

With the advent of molecular techniques, relationships within *Angraecum* have been explored in more detail over the last 15 years. The studies of Carlsward et al. (2006) on the phylogenetics of leafless angraecoids and of Micheneau et al. (2008) on the biogeography of Mascarene angraecoid orchids have shown the polyphyly of *Angraecum* and of at least five of the 19 recognised sections. In fact, African species of *Angraecum* belong to a group clearly distinct from that of members of the genus in Madagascar and the Indian Ocean islands (Micheneau et al. 2008). While investigating the diversification of the genus in Madagascar, Andriananjamanantsoa et al. (2016) also confirmed the polyphyly of *Angraecum* *s.l.* and of all *Angraecum* sections with representatives in Madagascar, with the sole exception of section Hadrangis Schltr. Agreeing with Micheneau et al. (2008), Szlachetko et al. (2013) indicated that most of the sectional arrangements within the genus are unnatural. Based on molecular and morphological data, Szlachetko et al. (2013) raised most of the sections, *sensu* Schlechter (1918) and Garay (1973), to the rank of genus, albeit with a different placement for some species. However, their sampling of DNA material lacked more than 3/4 of the species of *Angraecum* (only 53 of the 221 currently recognised species were included in their phylogeny) and several sections were unrepresented. In all, Szlachetko et al. (2013) recognised 18 genera that included species previously placed within *Angraecum*, 12 of which resulted from raising sections to the generic level, five of which were described as new genera and one of which involved resurrecting a genus previously placed in synonymy.

Szlachetko et al. (2013) established the genus *Pectinariella* Szlach., Mytnik & Grochocka to accommodate the species of Angraecum sect. Pectinaria Benth. *sensu* Garay (1973) since the sectional name was already occupied at the generic level in another family (*Pectinaria* Haworth, Apocynaceae). They circumscribed *Pectinariella* to include all members previously assigned to sect. Pectinaria from continental Africa and the Gulf of Guinea islands as well as those from the western Indian Ocean islands. However, their molecular study lacked material of the type species, *A. pectinatum* Thouars and included only five of the ten species of Angraecum sect. Pectinaria: four from Africa (*viz. A. doratophyllum* Summerh., *A. gabonense* Summerh., *A. pungens* Schltr. and *A. subulatum* Lindl.) and one from Madagascar (*A. dasycarpum* Schltr.).

Cribb (2014) suggested that part of Angraecum sect. Pectinaria might be separated from *Angraecum* *sensu stricto*, given that the generitype, *A. eburneum* Bory, was placed in the large Malagasy/Mascarene clade identified by Micheneau et al. (2008). The polyphyly of the section was confirmed by Simo-Droissart et al. (2013) based on sequence data from all five currently recognised species from Africa (the four mentioned above plus *A. atlanticum* Stévart & Droissart), along with three species from Madagascar and the Mascarene Islands. Confirming the results of Micheneau et al. (2008), Simo-Droissart et al. (2013) showed that the African members of A. sect. Pectinaria formed a well-supported clade that appears to be most closely related to A. sect. Dolabrifolia Pfitzer and is not sister to the Malagasy/Mascarene clade, which includes the type species of the section.

Based on these results, Simo-Droissart et al. (2013) suggested that it would be necessary to remove the African species from Angraecum sect. Pectinaria in order to maintain its monophyly. The generic name *Pectinariella* proposed by Szlachetko et al. (2013) cannot, however, be applied to these African species since its type, *P. pectinata* (Thouars) Szlach., Mytnik & Grochocka, belongs to the clade comprising Malagasy/Mascarene taxa. Following the treatments of Szlachetko and Romowicz (2007) and Szlachetko et al. (2013), which treat the taxa generally placed in A. sect. Dolabrifolia as a genus, *Dolabrifolia* (Pfitzer) Szlach. & Romowicz and, since the African members of their polyphyletic genus *Pectinariella* are sister to those of *Dolabrifolia*, they must also be recognised at the rank of genus.

Here we thus propose a new genus to accommodate the species from continental Africa and the Gulf of Guinea islands of *Pectinariella*.

## Taxonomy

### Afropectinariella

Taxon classificationPlantaeAsparagalesOrchidaceae

M.Simo & Stévart gen. nov.

urn:lsid:ipni.org:names:60476170-2

#### Type.

*Afropectinariella doratophylla* (Summerh.) M.Simo & Stévart [≡ *Angraecum doratophyllum* Summerh.].

#### Etymology.

The name of the genus is based on the geographic distribution of its five species, all of which occur in Africa and the generic name *Pectinariella* in which they were previously placed.

#### Diagnosis.

*Afropectinariella* resembles the related genera *Dolabrifolia* and *Pectinariella* in having a sessile ovary, i.e. without a pedicel and with a very short peduncle that is hardly developed, but differs from *Dolabrifolia* by its elongate leaves that are never compressed laterally (vs. imbricate and laterally compressed) and from *Pectinariella* by its transversely oval lip that is wider than long (vs. the lip longer that wide) and its occurrence in continental Africa and Gulf of Guinea islands (vs. Madagascar and adjacent islands).

#### Description.

Epiphytic herbs. Stem erect to pendent, branched and loosely leafy. Leaves fleshy, alternate and elongate, subulate or linear to oblong-ovate, apex acute to apiculate, petiole twisted. Inflorescences suberect and subsessile, in general 1-flowered, sometimes 2-flowered, borne along the stem or opposite a leaf, sheath brown. Flowers small, nearly sessile in the axils of the leaves, white and often scented. Floral bract one, amplexicaul, broadly ovate. Sepals and petals elliptic to obovate, apex subacute. Lip entire, ovate-triangular, ecallose, apex acute to apiculate. Spur ellipsoid or subcylindric, straight or slightly curved, sometimes hooked, with a wide mouth at the base of the lip, apex acute, often blunt or swollen in the apical half. Peduncle short, hardly developed. Pollinia 2, pyriform, often sessile on one or two viscidia, without a distinct stipe or shortly stipitate, rarely with a well-developed stipe.

*Afropectinariella* includes five species from continental Africa and the Gulf of Guinea Islands, one of which is endemic to São Tomé and Príncipe Islands. Four of these five species were placed in Angraecum sect. Pectinaria sensu Garay (1973) and, more recently, in *Pectinariella*, as originally circumscribed by Szlachetko et al. (2013). A detailed taxonomic treatment of these five species is presented in Simo-Droissart et al. (2014).

### Afropectinariella atlantica

Taxon classificationPlantaeAsparagalesOrchidaceae

(Stévart & Droissart) M.Simo & Stévart comb. nov.

urn:lsid:ipni.org:names:60476171-2

#### Basionym.

*Angraecum atlanticum* Stévart et al. (2010: 253). Type: Equatorial Guinea (Rio Muni). Monte Alén National Park: Engong inselberg, 5 km NW from Engong village, 01°37'25.8"N, 10°17'49.2"E, 1,100 m alt., 20 July 2001, *Stévart 1020* (holotype: BRLU!; isotypes: MO!, K!, WAG!).

### Afropectinariella doratophylla

Taxon classificationPlantaeAsparagalesOrchidaceae

(Summerh.) M.Simo & Stévart, comb. nov .

urn:lsid:ipni.org:names:60476172-2

#### Basionym.

*Angraecum doratophyllum* Summerhayes (1937: 465). Type: São Tomé and Príncipe (São Tomé Island) Vanhulst (Macambrará): virgin forest, 1,050–1,200 m alt., 5 November 1932, *Exell 254* (holotype: BM [BM000539167]! isotype: K [K000306497]!).

#### Homotypic synonym.

*Pectinariella doratophylla* (Summerh.) Szlach., Mytnik

& Grochocka

### Afropectinariella gabonensis

Taxon classificationPlantaeAsparagalesOrchidaceae

(Summerh.) M.Simo & Stévart comb. nov.

urn:lsid:ipni.org:names:60476173-2

#### Basionym.

*Angraecum gabonense* Summerhayes (1954: 587). Type: Gabon. Upper Ngounyé River, Nimalaba, N. E. of Les Echiras, 15 February 1927, *Le Testu 6384* (holotype: K [K000306496]! isotypes: BM [BM000539175]! P [P00388248]!).

#### Homotypic synonym.

*Pectinariella gabonense* (Summerh.) Szlach., Mytnik

& Grochocka

### Afropectinariella pungens

Taxon classificationPlantaeAsparagalesOrchidaceae

(Schltr.) M.Simo & Stévart comb. nov.

urn:lsid:ipni.org:names:60476174-2

#### Basionym.

*Angraecum pungens* Schlechter (1906: 163). Type: Cameroon. Man O’War Bay, auf Baümen bei Kriegschiffhafen, 03°57’N, 09°14’E, 27 September 1905, *Schlechter 15774* (holotype: B, destroyed; lectotype: K [K000107121]!, isolectotypes: BM [BM000539131]!, BR [BR000000642136]!).

#### Homotypic synonyms.

*Angraecopsis pungens* (Schltr.) Rice; *Pectinariella pungens* (Schltr.) Szlach., Mytnik & Grochocka

#### Heterotypic synonyms.

*Mystacydium arthrophyllum* Kraenzl.; *Angraecum arthrophyllum* (Kraenzl.) Schltr.

### Afropectinariella subulata

Taxon classificationPlantaeAsparagalesOrchidaceae

(Lindl.) M.Simo & Stévart comb. nov.

urn:lsid:ipni.org:names:77177891-1

#### Basionym.

*Angraecum subulatum* Lindley (1837: 206). Type: Nigeria. Nun River: s. d., *Barter 20125* (lectotype: K!, isolectotype: K!).

#### Homotypic synonyms.

*Epidorkis subulata* (Lindl.) Kuntze; *Listrostachys subulata* (Lindl.) Rchb.f.; *Pectinariella subulata* (Lindl.) Szlach., Mytnik & Grochocka

#### Heterotypic synonyms.

*Angraecum canaliculatum* De Wild.; *Listrostachys canaliculata* De Wild.

## Discussion

Although we have removed species from continental Africa and the Gulf of Guinea islands from *Pectinariella*, its delimitation remains open to further discussion. Garay (1973) included four species from Madagascar and the Mascarenes within Angraecum section Pectinaria (i.e. *A. dasycarpum*, *A. hermannii* (Cordem.) Schltr., *A. humblotianum* Schltr. and *A. pectinatum*). He regarded *A. pterophyllum* H.Perrier as a synonym of *A. hermannii* and placed *A. panicifolium* H.Perrier in Angraecum sect. Conchoglossum Schltr. In addition to the four species recognised by Garay (1973) as comprising section Pectinaria, Stewart et al. (2006) also included *A. panicifolium*. Micheneau et al. (2008) suggested that *A. hermannii* might best be placed in A. sect. Lemurangis Garay based on its morphological characters, which was followed by Cribb and Hermans (2009) and Verlynde et al. (2016). Cribb and Hermans (op. cit.) recognised *A. pterophyllum* as a distinct species and thus included five species within the section. As they followed the treatment of Garay (1973), Szlachetko et al. (2013) placed only four species from Madagascar and the Mascarenes within their newly described genus *Pectinariella* (*P. dasycarpa* (Schltr.) Szlach., Mytnik & Grochocka, *P. hermannii* (Cordem.) Szlach., Mytnik & Grochocka, *P. humblotiana* (Schltr.) Szlach., Mytnik & Grochocka and *P. pectinata*). In fact, these authors made no mention of *Angraecum pterophyllum*, but they did propose the combination *Angraecoides panicifolia* (H. Perrier) Szlach., Mytnik & Grochocka. Verlynde et al. (2016) described two new species of *Pectinariella* (*P. edmundi* Bosser ex Verlynde & Ramandimbisoa and *P. scroticalcar* Verlynde & Ramandimbisoa) and made the combination *P. pterophylla* (H.Perrier) Verlynde & Ramandimbisoa, thus recognising six species of *Pectinariella* from Madagascar and the Mascarenes. They did not mention *Angraecum panicifolium* or *Angraecoides panicifolia*, even though Simo-Droissart et al. (2013) placed it close to *Pectinariella pectinata*. *Pectinariella* thus comprises seven species, four of which have been included in published molecular phylogenetic studies. These seven species are found in Madagascar or neighbouring islands, including the type species, which occurs in La Réunion, Mauritius and the Comoros Islands.

In the studies of Simo-Droissart et al. (2013, 2016), species belonging to *Dolabrifolia* and *Afropectinariella* form two distinct, well-supported clades (bootstrap support (BS) = 100% and posterior probability (PP) = 1). The sister relationship of these two groups is also well supported by our Bayesian analysis (PP 1). Species assigned to *Pectinariella* also form a clade well supported in both analyses and are not sister to the clade comprising species of our new genus, *Afropectinariella*. The shared presence of elongate leaves that are never compressed, an ovary lacking a stalk and whose peduncle is scarcely developed appear to represent a convergence in *Pectinariella* and *Afropectinariella*. However, members of *Afropectinariella* possess a transversely oval lip that is wider than long, while those of *Pectinariella* have a lip that is longer that wide. Unpublished molecular data indicate that *P. edmundi* and *P. scroticalcar* are not sister to *P. pectinata*, suggesting that the genus, as circumscribed by Verlynde et al. (2016), may not be monophyletic. The fact that some Angraecum species assigned to section Pseudojumellea Schltr. are placed with *P. pectinata* in molecular phylogenies (see Micheneau et al. 2008) indicates that further studies will be needed to assess the monophyly of *Pectinariella*.

## Supplementary Material

XML Treatment for Afropectinariella

XML Treatment for Afropectinariella atlantica

XML Treatment for Afropectinariella doratophylla

XML Treatment for Afropectinariella gabonensis

XML Treatment for Afropectinariella pungens

XML Treatment for Afropectinariella subulata
